# Enhancing Dementia Caregivers’ Emotional Coping in an Intergenerational Expressive Arts Based Therapy Program

**DOI:** 10.1093/geroni/igaa057.1150

**Published:** 2020-12-16

**Authors:** Keisha Carden, Rebecca Allen, Katherine Ramos, Daniel Potts, Keisha Ivey

**Affiliations:** 1 University of Alabama, Tuscaloosa, Alabama, United States; 2 Alabama Research Institute on Aging, Tuscaloosa, Alabama, United States; 3 Duke University School of Medicine, Raleigh, North Carolina, United States; 4 Tuscaloosa VA Medical Center, Tuscaloosa, Alabama, United States

## Abstract

Dementia caregivers often experience elevated levels of stress and are at increased risk for psychological disorders. Recent research has implicated emotional avoidance, psychological inflexibility, and related processes in the development and maintenance of psychopathology. Addressing these processes is essential for improving caregiver mental health. This pilot study examined changes in emotional avoidance, psychological inflexibility, and anticipatory grief among familial caregivers of individuals with dementia enrolled in an intergenerational expressive arts program (Bringing Art to Life). Caregivers were an average age of 63.32 (11 NHW, 8 AA; 13 Women, 6 Men). On average, caregivers’ emotional avoidance and psychological inflexibility (M = 19.63, SE = 2.16) decreased significantly after participating in the program (M = 15.06, SE = 1.80). This difference, -4.57, BCa 95% CI [1.82, 7.13], was significant, t(15) = 3.81, p = .002, and represented a medium-sized effect, d = 0.70. Overall experience of anticipatory grief (M = 51.88, SE = 3.26) also decreased (M = 48.00, SE = 2.79). More specifically, caregivers’ reported sadness and loneliness (M = 17.63, SE = 1.37) decreased significantly (M = 14.5, SE = 0.95), BCa 95% CI [1.25, 5.00], t (15) = 3.21, p = 006, and represented a medium-sized effect, d = 0.64. These findings illuminate novel ways in which interventions might both target underpinnings of the caregiver stress process and improve the dyadic relationship. Specific intervention elements are explored and recommendations are made for future intervention development and implementation as well as measurement based outcome tracking.

